# Effect of Solvent Hydrogen‐Bonding and Catalyst Pore Size on Catalytic Oxidation of Benzylic Alcohols

**DOI:** 10.1002/cphc.70471

**Published:** 2026-07-31

**Authors:** Dian Jing, Justin M. Notestein

**Affiliations:** ^1^ Department of Chemical and Biological Engineering Northwestern University Evanston Illinois USA

**Keywords:** alcohol, alcohol oxidation, catalysis, catalytic oxidation, chemistry, hydrogen peroxide, reaction rate, steric effects, sulfolane

## Abstract

Control over selective oxidation rates remain a key challenge in industrial chemicals production. Here, we demonstrate how liquid phase alcohol oxidation is a complex interplay among solvent, reactant, catalyst porosity, and local active site environment. Specifically, we study the impact of sulfolane versus acetonitrile solvents on rates of catalytic oxidation of benzylic alcohols to the corresponding ketone using hydrogen peroxide (H_2_O_2_) over Ti‐based heterogeneous catalysts of different local steric environments. The polar, H‐bonding sulfolane enhances the reaction rate in wide pore materials, presumably by promoting hydrogen transfer steps. However, in sulfolane, the reaction rate also decreases as the local environment around the Ti active site becomes more sterically congested, either by decreasing catalyst pore size or by increasing reactant bulk. This is assigned to an effect of the bulky hydrogen‐bonding complex formed between reactant alcohol and sulfolane that introduces transport limitations. Finally, large‐pore Ti–SiO_2_ catalysts modified with additional SiO_2_ near the active sites were able to further tune reaction rates in these two solvents by a combination of effects induced in the immediate pore environment. Better understanding of size‐ and solvent‐dependent transport and kinetic effects should allow for improved control over the rate of selective oxidation reactions.

## Graphical abstract

Selective benzylic alcohol oxidation using hydrogen peroxide (H_2_O_2_) is catalyzed by an array of Ti‐silica materials. Sulfolane solvent strongly interacts with the reactant and enhances rates versus acetonitrile solvent in wide pore materials, but negative transport effects dominate in smaller‐pore materials. Partially overcoated catalysts stabilize the active site and give high rates in sulfolane, presenting a new route to tune activity.

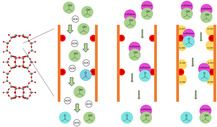

## 1 Introduction

The rate of alcohol oxidation is crucial in many chemical production systems, and fine‐tuning this rate is essential for achieving the desired conversion and selectivity. While faster oxidation may improve reactor utilization, it may conversely be necessary to moderate reaction rates in order to control reaction selectivity, prevent overoxidation, or minimize thermal runaway. One model system where these effects have been investigated is the benzylic oxidation of alkyl aromatics with transition metal catalysts and hydrogen peroxide as the oxidant [1, 6]. In these oxidation reactions, benzylic alcohols are first formed and then transformed into the corresponding ketones. Since the alcohol products are often more active toward oxidation than the alkyl aromatics, these oxidation reactions tend to yield the more thermodynamically stable ketone products, especially as conversion increases [7, 9]. This is an example of a system where one may want to slow alcohol oxidation in order to select for the intermediate oxidation product. In a related system consisting of the series oxidation reaction of benzene into phenol and subsequently into benzoquinones [10], it was shown that phenol selectivity was enhanced by using sulfolane solvent combined with porous catalysts. It was argued that the strongly hydrogen‐bonding nature of sulfolane was the origin of these effects [11, 12]. Specifically, it was proposed that sulfolane formed a bulky hydrogen‐bonding complex with phenol, increasing the effective steric hindrance of phenol, inhibiting the diffusion of the intermediate phenol into the porous catalyst, and thus improving its selectivity. Sulfolane is well known to form strong interactions with water and a variety of alcohols, leading to complex solution behavior [13]. Sulfolane is also used as a molecular crowding agent in electrolyte solutions because of how it alters the H‐bond network [14].

In this article, we investigate the interplay of reactant size, solvent effects, and catalyst structure (pore and particle sizes) on the reaction and diffusion kinetics of benzylic alcohol oxidation. To this end, we study the oxidation of ethylbenzyl alcohol and derivatives into the corresponding ketones, using porous Ti–SiO_2_ catalysts and H_2_O_2_ as a mild oxidant requiring catalytic activation [15, 16]. We investigated the effect of hydrogen‐bonding sulfolane solvent in comparison to the commonly used, chemically stable, nonhydrogen bonding acetonitrile solvent which does not participate directly in the reaction [12]. Note that the reaction conditions of this study are far from those where acetonitrile is oxidized to peroxycarboximidic acid and plays a direct role in the reaction network [17]. Conversely, neither acetonitrile nor sulfolane would be expected to interact with the active sites through anything other than Lewis acid–Lewis base adducts. We also examined a range of Ti–SiO_2_ catalysts with different pore size distributions (microporous TS‐1 and Ti‐BEA, and three macroporous samples Ti–SiO_2_‐A, Ti–SiO_2_‐B, and Ti–SiO_2_‐C), pore hydrophobicity (hydrophilic vs. hydrophobic Ti‐beta), and local Ti environments (macroporous Ti–SiO_2_ overcoated with additional SiO_2_). In some cases, different particles sizes were used to provide further evidence for transport effects. Here, the sulfolane solvent generally promotes high rates of oxidation, consistent with prior reports that hydrogen‐bonding complexes facilitate proton transfer during the oxidation reaction [18, 23], consequently enhancing the intrinsic rate of oxidation. However, we observe that the rate enhancement relative to acetonitrile solvent is attenuated in confined pore spaces.

There has been significant interest in recent years on how solvent, hydrogen bonding, and pore confinement interact to govern transition state stabilization [19]. For example, previous studies on epoxidation reactions in confined cavities, such as in BEA and MFI zeolites, suggested that the interplay between local hydrophilicity or SiOH density and local confinement could affect the rate of reaction by controlling solvent rearrangement at the transition state [21, 24, 26]. Confinement alone can have a significant impact on apparent rates by altering the strength of reactant adsorption near active sites, as induced by zeolite micropores or added oxide near the active site [27, 28]. Finally, high densities of hydroxyls near active sites can directly influence the kinetics of proton shuttling inherent to H_2_O_2_ activation [29, 31]. However, it is somewhat risky to generalize away from examined cases, and the effect has been most studied in H_2_O_2_ activation reactions for epoxidation, where the alkene reactant itself does not engage in significant H‐bond forming interactions. In the work carried out here, it appears that the bulky complex resulting from interactions between alcohol reactants and sulfone solvent effectively introduces steric hindrance to the reactant, leading to decreased rates of diffusion within porous Ti–SiO_2_ structures, offsetting any intrinsic stabilization. Because solvent interactions are expected to vary significantly between hydrocarbon and alcohol reactants, these results suggest that combining pore confinement, solvent effects, and local active site effects may be an effective route to tune oxidation catalysis rates of oxygenated species.

## 2 Methods

### 2.1 Catalyst Synthesis

All catalysts used in this work were synthesized for previous studies of different reactions. Macroporous Ti–SiO_2_ catalysts were synthesized by grafting bulky Ti‐containing precursors onto three commercial SiO_2_ samples, as reported previously [28, 32]. The Ti precursor was commercial Cp*TiCl_3_ (Strem Chemicals, 98%) for Ti–SiO_2_‐A and Ti–SiO_2_‐B, or monomethoxy‐tert‐butylcalix [4]arene titanium chloride (mmCalixTiCl) synthesized from 1,3‐dimethoxy‐tert‐butylcalix [4]arene [33] and TiCl_4_ (Strem Chemicals, 99%) for Ti–SiO_2_‐C.

SiO_2_‐A (Alfa‐Aesar, labeled as 15 nm pore diameter, 100–200 mesh, 350–400 m^2^/g), SiO_2_‐B (Alfa‐Aesar, labeled as 9 nm pore diameter, 100–200 mesh, ~375 m^2^/g), or SiO_2_‐C (Selecto Scientific, labeled as 5.4 nm average pore diameter, 32–62 μm particle size, 570 m^2^/g) were dried overnight at 120°C–190°C under vacuum and then transferred to a round‐bottom flask under N_2_. Freshly distilled toluene (Sigma‐Aldrich, ACS Reagent, ≥99.5%) and the Ti precursor were added in amounts equivalent to 0.2–0.5 Ti/nm^2^ of the support. The mixture was stirred with a Teflon stir bar under N_2_ overnight until the silica support acquired the color of the Ti precursor. The Cp*TiCl_3_ grafting was carried out at room temperature, while the mmCalixTiCl was grafted at reflux. The catalyst was separated by vacuum filtration, washed with toluene until the washes ran clear, and was dried under vacuum filtration. These samples could be stored for several years in a desiccator in this form. Before use, catalysts were calcined in static air at 550°C for 6–8 h with a ramp rate of 10°C/min until they became bleached white powders of Ti–SiO_2_.

Overcoated SiO_2_@(Ti–SiO_2_) materials were synthesized following a previously published procedure [28]. For Partially Overcoated catalysts (2 cPO and 10 cPO), as‐synthesized Cp*Ti–SiO_2_‐B was used as overcoat template, whereas for Fully Overcoated catalysts (2 cFO and 10 cFO), calcined Ti–SiO_2_‐B was used. For the overcoat, 2 g of either template, 188 mL of ethanol (Decon Labs, 200 proof, ≥99%), and 28 mL of NH_4_OH (Macron Fine Chemicals, ≥99.9%) were added to a 500 mL HDPE bottle. For each cycle of overcoating, the bottle was sonicated for 30 min to disperse the template powders, 0.65 mL of tetraethylorthosilicate (TEOS, Sigma‐Aldrich, ≥99.0%) was added to the mixture, and the mixture was shaken at 200 rpm for 1 h on a gyratory plate. After 2 or 10 cycles of overcoat, the catalysts were separated by vacuum filtration, rinsed with ethanol and then hexane, and dried under vacuum filtration. The catalysts with 2 cycles of overcoat were labeled 2 cPO or 2 cFO and those with 10 cycles of overcoat were labeled 10 cPO or 10 cFO. The catalysts were used in reaction after calcination in static air at 550°C for 6–8 h with a ramp rate of 10°C/min until they became bleach white powders.

Hydrophilic Ti‐BEA and hydrophobic Ti‐BEA were gifts of the Flaherty laboratory, and were synthesized following the procedures in previous studies [24, 26]. Commercially available Al‐BEA with a Si/Al ratio of 12.5 (250) was used to prepare hydrophilic (hydrophobic) Ti‐BEA. The parent Al‐BEA was first treated to remove aluminum atoms under refluxing HNO_3_ (Macron Chemicals, 68–70 wt.%) for 18 h. The zeolite was separated by vacuum filtration, rinsed with H_2_O (17.8 MΩ·cm, 50 cm^3^/g), and dried by heating under a continuous flow of air (Airgas, Ultra‐zero grade; 100 cm^3^/min) at 550°C for 6 h with a ramp rate of 5°C/min to form Si‐BEA with Si/Al ratios >1400. To incorporate Ti atoms, Si‐BEA was suspended in CH_2_Cl_2_ (Fisher Chemicals, ACS grade) and refluxed with an appropriate amount of TiCl_4_ (Sigma‐Aldrich, 99.9%) for 4 h. The catalyst was separated by removing volatile components via rotary evaporation and calcined in static air at 550°C for 6–8 h with a ramp rate of 10°C/min before being used in reaction.

Titanium silicalite‐1 (Ti‐MFI or TS‐1) was a historical donation by Eni Chemical. The titanium loading of the catalyst was provided by the manufacturer to be 1.8% wt. The catalyst was calcined in static air at 550°C for 6–8 h with 10°C/min ramp rate before used in reaction.

### 2.2 Catalyst Characterization

The surface area and pore size distribution of the Ti–SiO_2_‐B catalyst and the overcoated SiO_2_@(Ti–SiO_2_) catalysts are as reported in previous work [28]. The values were determined from N_2_ physisorption performed on Micromeritics ASAP 2010 instrument after the catalysts were dried under vacuum at 450°C overnight. The surface area of all other catalysts and the pore size distribution of Ti–SiO_2_‐A and Ti–SiO_2_‐C were obtained by N_2_ physisorption performed on Micromeritics 3Flex BET instrument after the catalysts were dried under vacuum at 100°C overnight. N_2_ physisorption was performed at ‒196°C. The total surface area was determined by the Rouquerol‐modified‐Brunauer–Emmett–Teller (BET) method on the adsorption branch of the isotherm and the pore size distribution was determined by the BJH method on the desorption branch of the isotherm. For TS‐1 and Ti‐BEA catalysts, the pore sizes were crystallographically defined.

The Ti content for Ti–SiO_2_‐B catalyst, the overcoated SiO_2_@(Ti–SiO_2_) catalysts, Ti–SiO_2_‐C catalyst, hydrophilic Ti‐BEA, hydrophobic Ti‐BEA, and TS‐1 was either reported in previous works [28] or labeled by the producer. The Ti content for Ti–SiO_2_‐A catalyst was determined by thermogravimetric analysis (TGA). Approximately 49 mg of as‐synthesized Cp*Ti–SiO_2_‐A catalyst was added to the TGA instrument. Under a gas flow of 90.0 mL/min O_2_ and 10.0 mL/min N_2_, the temperature of the sample was increased from 5°C to 800°C with a ramp rate of 10°C/min and held for 10 min at 800°C. The weight of the sample before and after the process was obtained to calculate the amount of Cp* lost and the corresponding Ti content of the sample.

Edge energies of all catalysts were measured by diffuse reflectance UV–vis (DRUV–vis) spectra on freshly calcined catalysts. Total reflectance of each material was measured using Shimadzu UV‐3600 Plus UV–vis–NIR spectrophotometer with Harrick Praying Mantis diffuse reflectance accessory. The background spectrum was obtained with polytetrafluoroethylene (PTFE, Sigma‐Aldrich, 35 μm particle size). The edge energy of each catalyst was determined from the x‐intercept of the linear portion of the indirect transition Tauc plot of the spectra according to established method [34].

The particle size of all catalysts were measured by scanning electron microscopy (SEM) with freshly calcined catalysts. Around 1 mg of each catalyst was first suspended in 1 mL of ethanol and spin‐coated onto a silica wafer. The wafer was then plasma coated with osmium to make the surface conductive. SEM images of each sample were taken by an EPIC SEM Hitachi S‐3400 instrument and the diameter of a representative particle was measured directly on the SEM images.

### 2.3 Measurement of Rates and Reactant Order of Alcohol Oxidation Reaction

Alcohol oxidation reactions were performed in sealed septum vials. For each reaction, 10 mL of solvent, either acetonitrile (Fisher Chemical, HPLC, ≥99.9%) or sulfolane (Thermo Scientific Chemicals, 99%), and 40–200 mg of the catalyst were added to the reactor. Reactants included liquid 1‐phenylethanol (Sigma‐Aldrich, 98%) and 1‐(p‐tolyl)ethanol (Sigma‐Aldrich, ≥97%) and solid 1‐(2‐naphthyl)ethanol (TCI America, ≥98%). The mixture was heated to the reaction temperature of 80°C and shaken at 500 rpm on a shaker plate for 10–30 min to thermally equilibrate. The oxidation reaction was initiated by injecting aqueous H_2_O_2_ (Fisher Chemical, 30 wt.%) through the septum into the reactor. The base case scenario had a 1‐phenylethanol concentration of 0.62 M, a 1‐(p‐tolyl)ethanol concentration of 0.54 M, or a 1‐(2‐naphthyl)ethanol concentration of 0.48 M. The base case amount of H_2_O_2_ was 2 mL, corresponding to a final concentration of 1.5 M. The reader is cautioned to use fresh or freshly purified reagents and solvents if replicating this work. Background oxidation or decomposition of some of these species can react with H_2_O_2_ in our experience, leading to spurious results.

Aliquots of 0.05–0.1 mL were taken from the reactors and mixed with 0.5–1 mL d‐chloroform (Thermal Scientific Chemicals, 99.8%). ^1^H NMR spectra of the samples were obtained on a 400 MHz Bruker Avance III HD Nanobay system at room temperature. 32 scans were used for each sample with the D1 delay set to 5 s. The following peaks were integrated to quantify the concentration of each reactant or product: quartet at 4.83 ppm for 1‐phenylethanol, doublet at 7.91 ppm for acetophenone, quartet at 4.83 ppm for 1‐(p‐tolyl)ethanol, doublet at 7.83 ppm for 4′‐methylacetophenone (oxidation product of 1‐(p‐tolyl)ethanol), quartet at 5.07 ppm for 1‐(2‐naphthyl)ethanol, and singlet at 8.48 ppm for 2‐acetylnaphthalene (oxidation product of 1‐(2‐naphthyl)ethanol). Example NMR spectra and detailed peak identifications for all alcohol and ketone are provided in Supporting Information (Figures S5–S7). From each NMR spectrum, the relative area of the integrated peaks from the alcohol and the corresponding ketone was determined and compared with that of standard solutions, which were made with authentic products acetophenone (Fisher Chemical, 99%), 4′‐methylacetophenone (Thermo Scientific Chemicals, 96%), or 2‐acetylnaphthalene (Thermal Scientific Chemicals, 99%) and the corresponding alcohol, to obtain conversion. Since no side products were identified from the NMR spectra, mole balance between the initial alcohol reactant and the total alcohol and ketone in the reactor was assumed. Reaction turnover was then calculated as the product of conversion and the initial moles of reactant, divided by the catalyst mass and its Ti loading. These catalysts are known to be present primarily as single atoms, with intrinsic dispersions >75% and near 100% dispersion for many systems [28].

**FIGURE 1:**
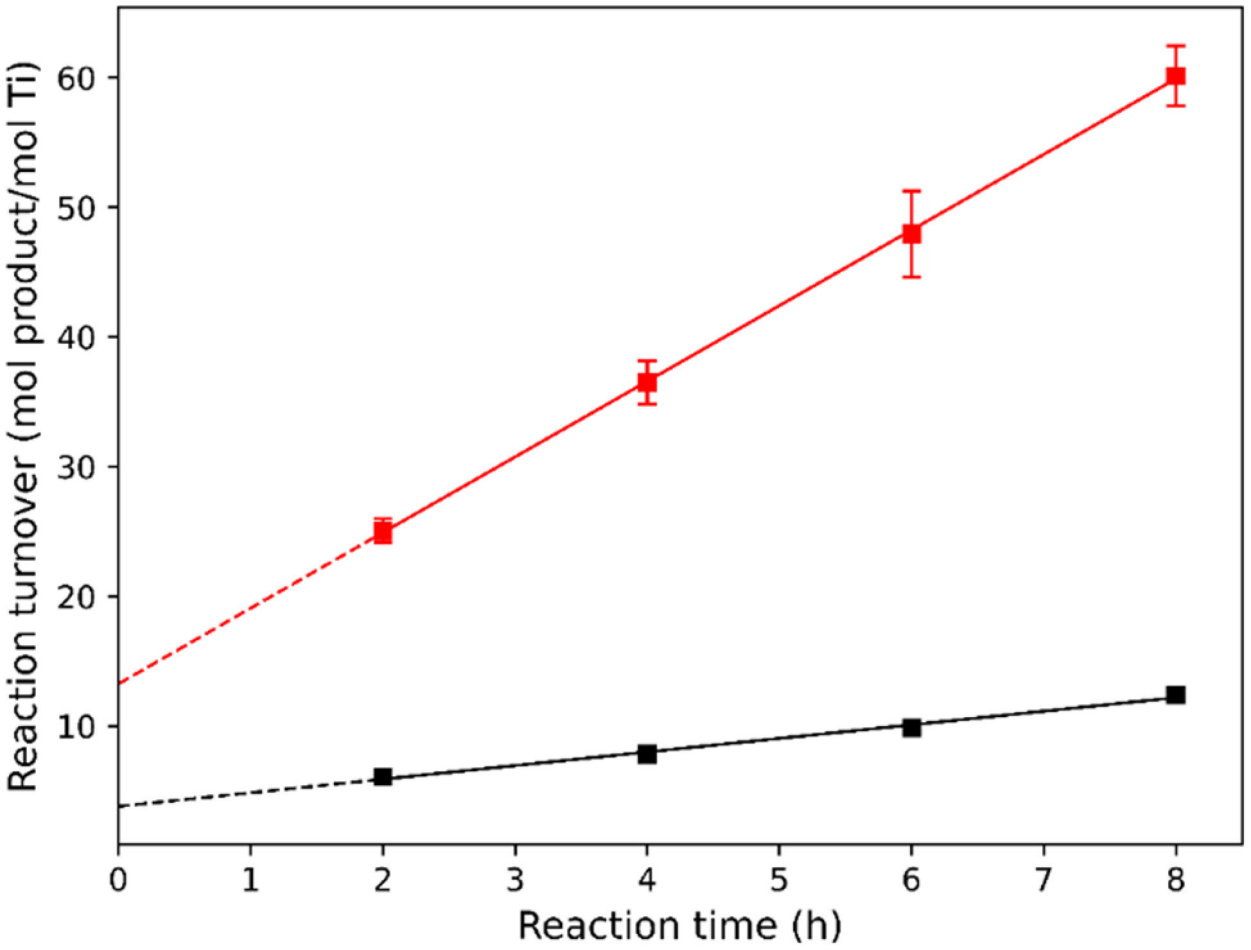
Representative plots of 1‐phenylethanol turnover as a function of reaction time with acetonitrile solvent (black) and sulfolane solvent (red). Linear regression fitted between 2 and 8 h of reaction time (solid line), with interpolation between 0 and 2 h (dashed line). The slopes of the regression lines correspond to the initial turnover rate. The nonzero y‐intercept at zero time reflects the reaction initiation effect. Error bars represent one standard error of the sample mean. Conditions: 80°C, 90 mg of Ti–SiO_2_‐A catalyst (28 μmol Ti), 8.0 mmol 1‐phenylethanol, 20 mmol H_2_O_2_, 10 mL solvent, shaking at 500 rpm.

For standard reactions, aliquots were taken at 2, 4, 6, and 8 h after the injection of H_2_O_2_, and the conversion at each 2‐h time interval was calculated from NMR analysis. The sampled aliquots accounted for approximately 1% of the reaction mixture, indicated minimal effect on the measured reaction rates for subsequent samples. The typical conversion after 8 h of reaction was 3%–14%. The conversion was deliberately kept low such that the initial turnover rate could be determined by the slope of linear regression line on the plot of reaction turnover against reaction time between 2 and 8 h. The reaction data in the first 2 h were not used in order to eliminate any effects of the approach to catalytic steady state. For all reaction conditions, the experiments were repeated at least three times and the error bars associated with each reaction turnover data point were indicative of one standard deviation. A plot of reaction turnover against reaction time for a representative condition was shown in Figure 1. The R^2^ value of all such linear regression lines were larger than 0.9, suggesting that the choice of linear regression was valid. The typical initial turnover rate was 1.0–15 mol product/mol Ti/h. To obtain the apparent reaction order of 1‐phenylethanol and H_2_O_2_, the concentration of 1‐phenylethanol was varied from 0.12 to 0.62 M and that of H_2_O_2_ was varied from 0.05 to 0.48 M separately. Aliquots were taken at 5 min and 2 h after initiation and analyzed with NMR to determine the initial turnover rates.

## 3 Results and Discussion

### 3.1 Catalyst Properties

This study utilized catalysts developed in prior work or obtained from collaborators. The materials are intended to be a representative cross‐section of Ti‐based catalysts, and we are not advocating here for one synthesis method over another. A summary of catalyst characterization data is given in Table 1. N_2_ physisorption was used to assess the surface area and pore diameter distribution of each catalyst (isotherms in Figure S3). We note that the average pore diameter of SiO_2_‐A and SiO_2_‐C differed from that stated by the manufacturer; we used our isotherms. Zeolite materials Ti‐BEA and TS‐1 list the crystallographically defined cage size. Pore size distributions for Ti–SiO_2_‐A, Ti–SiO_2_‐C, Ti‐BEA, and TS‐1 are given in Figure S4. The data for Ti–SiO_2_‐B and overcoated SiO_2_@(Ti–SiO_2_) materials were reported in a previous publication [28].

**TABLE 1:** Summary of catalyst characterizations.

Catalyst	Ti loading			Edge^a^	S.A.^b^	Pore diameter	Particle size
	(mmol/g)	(Ti/nm^2^)	(wt %)	(eV)	(m^2^/g)	(nm)	(μm)^c^
Ti–SiO_2_‐A	0.31	0.52	1.5^e^	3.5	356	9.0^g^	50
Ti–SiO_2_‐C	0.16	0.27	0.78^f^	3.4	363	9.2^g^	150
Hydrophilic BEA	0.063	0.061	0.30^f^	3.7	612	0.67^h^	0.5
Hydrophobic BEA	0.063	0.070	0.30^f^	3.7	540	0.67^h^	0.5
TS‐1	0.38	0.62	1.8^f^	3.3	364	0.50^h^	1
Ti–SiO_2_‐B^d^	0.13	0.21^i^	0.63	4.0 → 3.0	374^i^	9.0^i^	50
2 cFO	0.11	0.21^i^	0.54	4.1 → 4.0	326^i^	8.5^i^	50
10 cFO	0.07	0.24^i^	0.34	4.1 → 4.0	178^i^	8.1^i^	50
2 cPO	0.11	0.21^i^	0.54	4.2 → 3.9	324^i^	8.3^i^	50
10 cPO	0.07	0.24^i^	0.34	4.2 → 3.8	173^i^	7.9^i^	50

a From DRUV–vis and the x‐intercept of the indirect Tauc plot shown in Figures S1 and S2. Two values indicate measurements taken after 4 years storage in a dessicator.

b From the adsorption branch of the N_2_ physisorption isotherm shown in Figure S3.

c From direct measurement on SEM images.

d Material not used in further studies due to clustering of titanium atoms.

e From TGA measurements.

f Provided by catalyst manufacturer.

g From applying BJH method on the desorption branch of N_2_ physisorption isotherm.

h Crystallographically defined.

i Taken from previously published work [28].

The edge energy from DRUV–vis spectra provide information on the aggregation of titanium atoms on the surface of SiO_2_ template. The edge energies for Ti–SiO_2_‐A, Ti–SiO_2_‐C, hydrophilic Ti‐BEA, hydrophobic Ti‐BEA, and TS‐1 catalysts all indicated some—but not severe—aggregation [27]. It is interesting to note that for Ti–SiO_2_‐B and overcoated SiO_2_@(Ti–SiO_2_) materials, we have DRUV–vis spectra of the same batch of materials as‐synthesized and after storage in a desiccator for 4–5 years. In the older measurements, the edge energies of all five materials were between 4.0 and 4.2 eV, which indicated highly dispersed O_3_TiOH active sites. In the recent measurements, the overcoated materials continued to maintain a high level of dispersion with the edge energies between 3.8 and 4.0 eV. However, the edge energy of nonovercoated Ti–SiO_2_‐B catalyst decreased to 3.0 eV, indicating TiOx clustering. Such clustering was formed by the migration of titanium atoms via slow hydrolysis reactions over the period of over 4 years. These results showed that the overcoated silica layers around titanium active sites served as barriers against the migration of titanium atoms and prevented the aggregation of titanium over time. Without these overcoat layers, the catalytic activity of Ti–SiO_2_‐B catalyst was greatly reduced due to the migration and clustering of titanium atoms. Indeed, when the Ti–SiO_2_‐B material was used to catalyze 1‐phenylethanol oxidation reaction in this study, the reaction rate was negligible and comparable to that observed in control reactions without catalyst. In the absence of catalyst and at the conditions of Figure 1, reactions were <25% of the rate of that of Ti–SiO_2_‐A in acetonitrile, otherwise the slowest material. Without catalyst, no statistically significant reaction was recorded in sulfolane.

### 3.2 Mechanism and Order of Alcohol Oxidation Reaction

**SCHEME 1:**
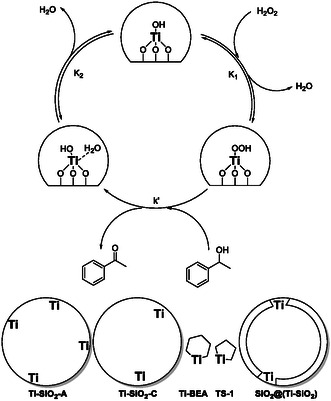
Proposed mechanism of 1‐phenylethanol oxidation reaction with three elementary steps: activation of pore confined titanium catalytic site by H_2_O_2_, oxidation of 1‐phenylethanol into acetophenone by the activated catalytic site, and desorption of water product from the titanium site. *K*_1_ and *K*_2_ are the equilibrium constant of activation and desorption steps, respectively, and k′ is the rate constant for the oxidation step. 1‐Phenylethanol is used as an example in this scheme and the mechanism for 1‐(p‐tolyl)ethanol and 1‐(2‐naphthyl)ethanol oxidation is proposed to follow the same mechanism. At bottom are representations of the pore environment of each of the classes of materials tested in this article.

In this study, the oxidation reaction of benzylic alcohols into the corresponding secondary ketone was investigated. In order of increasing size, the three alcohol reactants used were 1‐phenylethanol, 1‐(p‐tolyl)ethanol, and 1‐(2‐naphthyl)ethanol. The reaction was catalyzed by various titania‐on‐silica catalysts discussed above and carried out in acetonitrile or sulfolane solvent. The oxidizing agent was hydrogen peroxide, and no products other than the corresponding ketones were observed at levels detectable by NMR. The reaction mechanism was simply modeled to contain three elementary steps: activation of H_2_O_2_ over bare TiOH to form TiOOH sites, oxidation reaction on the activated site, and desorption of water and ketone product from the titanium site (Scheme 1). The alcohol reactant, the ketone product, water, and the solvent could also, in principle, compete for active sites.

**FIGURE 2:**
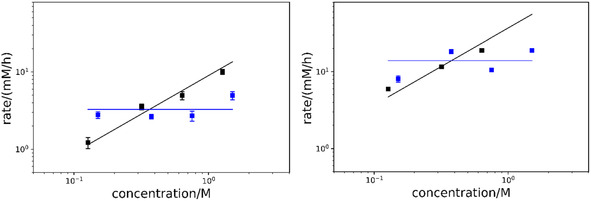
Plots of initial oxidation reaction rate against the initial concentration of 1‐phenylethanol (black) and the initial concentration of H_2_O_2_ (blue) with acetonitrile solvent (left panel) and sulfolane solvent (right panel). Over the range of concentration of interest, the reaction is approximately first order in the alcohol and zeroth order in H_2_O_2_ in both solvents. The dotted lines on the left panel were $rate=9.0*10^{-3}[\text{alcohol}]\;h^{-1}$ (black) and rate=3.3 mM/h (blue); the dotted lines on the right panel were $rate=37*10^{-3}[\text{alcohol}]\;h^{-1}$ (black) and rate=14 mM/h (blue). Error bars represent one standard error of the sample mean. Conditions: 80°C, 82 mg of Ti–SiO_2_‐C catalyst (13 μmol Ti), 8.0 mmol 1‐phenylethanol, 20 mmol H_2_O_2_, 10 mL solvent, shaking at 500 rpm.

The reaction order of alcohol and H_2_O_2_ was investigated using 1‐phenylethanol and Ti–SiO_2_‐C catalyst as a representative system. The reaction order study was carried out for both acetonitrile and sulfolane solvent. The concentrations of 1‐phenylethanol and H_2_O_2_ were independently varied over 0.13–1.3 M and 0.15–1.5 M, respectively. The initial reaction rate was measured and plotted against 1‐phenylethanol or H_2_O_2_ concentration on a log–log scale (Figure 2). The data approached first order in 1‐phenylethanol and zeroth order in H_2_O_2_ over the range of concentrations of interest. Specifically, the reaction orders were determined to be 0.87 ± 0.13 in 1‐phenylethanol and 0.22 ± 0.20 in H_2_O_2_ for acetonitrile solvent. For sulfolane solvent, they were 0.72 ± 0.03 in 1‐phenylethanol (two‐sided Student‐t test for first order gives *p* = 0.04) and 0.27 ± 0.35 in H_2_O_2_. Reported uncertainties represent 68% confidence intervals obtained from weighted linear fits with an added common variance term chosen such that the reduced χ^2^ equaled unity. These reaction orders are typical [4, 20, 35, 36], and they were the same for both acetonitrile and sulfolane solvents. These results confirm that it is meaningful to directly compare the apparent reaction rates (or rate constants) in both solvents.

These data are consistent with the assumptions that the most abundant surface intermediate is a TiOOH species with little competition from reactant, product, water, or solvent adsorption, that the rate‐limiting step of the reaction is the oxidation of the alcohol reactant into the ketone product by TiOOH species [36, 37], and that H_2_O_2_ is in excess. These data are also consistent with rate limiting transport of the alcohol to the active site. For either scenario, the turnover rate is modeled as

turnover rate=k[Alcohol]

Although the apparent rate orders alone do not provide specific mechanistic insight in this system, they do permit the use of first‐order rate constants to compare systems. In the former case, the reaction rate constant *k* is equal to the rate constant of the rate‐limiting step (*k′*) in units of $\frac{L}{mol\;Ti*h}$. In the latter case, $k=\frac{4\pi R^{2}\sqrt{k^{''}D}}{mol\;Ti}$, where *R* is the average radius of a catalyst particle, *k″* equals to the turnover rate constant at the active TiOOH site multiplied with the volume density of TiOOH sites, and *D* is the diffusion coefficient of reactant inside the catalyst particle.

### 3.3 The Effects of Pore Size in Hydrogen‐Bonding Solvent

The first goal of this work was to study the effects of hydrogen‐bonding solvent on the apparent rate of oxidation reaction of secondary alcohols. This information may be useful, for example, in controlling selectivity in series oxidation reactions.

**SCHEME 2:**
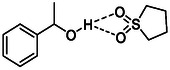
Illustration of sulfolane–alcohol complex. Complex formed by alcohol and sulfolane through hydrogen‐bonding. Dashed line denotes hydrogen bonds. This complex was hypothesized to decrease the effective diffusivity of the alcohol inside the catalyst pores. 1‐Phenylethanol is used as an example in this scheme.

Hydrogen‐bonded complexes are commonly formed between alcohols and appropriate solvents (e.g., Scheme 2) [11, 12, 22]. In addition to possible effects on the elementary steps of the reaction, these complexes have a larger effective kinetic diameter than the bare alcohol reactant and hence is more sensitive to diffusion and size‐exclusion effects inside the porous structures of heterogeneous catalysts. Here, sulfolane was picked as the strongly hydrogen‐bonding and bulky solvent as discussed in the introduction, and it was compared to a nonhydrogen‐bonding and small solvent as control. The nonhydrogen‐bonding solvent was chosen to be acetonitrile because it is safe under our reaction conditions, will not directly react with the active site or the product, and it is commonly used. Regardless of the catalyst, H‐bonding solvents have been previously observed to weaken the alcohol O─H bond, lowering the apparent activation energy required, and increasing observed rates for oxidation [22].

Here, the reaction of 1‐phenylethanol and Ti–SiO_2_‐A catalyst is taken as the reference case, as it is expected to have the least steric congestion within the wide catalyst pores. The Weisz–Prater parameter under this condition was calculated to be about 0.27 with acetonitrile solvent and 1.5 with sulfolane solvent, according to $\frac{kR^{2}}{D}$, where *k* is the rate constant and *R* is the catalyst particle radius in Table 1. The diffusion coefficient, *D*, was assumed to be 10^−12 ^m^2^/s for both solvents, based on published values for ethylbenzene and xylenes [38] or for propanediol [39] in similar materials. The single‐particle density of the catalyst was assumed to be 0.75 g/cm^3^ based on typical values of total pore volume obtained by N_2_ physisorption. Since the effect of diffusion was expected to be more severe for all other reaction conditions, this indicated that diffusion may impact the apparent reaction rate for all catalysts under the conditions of this study.

Oxidation reactions were performed with benzylic alcohols of increasing size (1‐phenylethanol, 1‐(p‐tolyl)ethanol, 1‐(2‐naphthyl)ethanol) and catalysts of decreasing pore diameter (Ti–SiO_2_‐A, Ti–SiO_2_‐C, Ti‐BEA, TS‐1). All the reactions performed showed linear relationships between turnover and reaction time, as seen in Figures S8–S10, which is in agreement with our observation that the oxidation reaction was first order in alcohol and was performed at low conversion. Rate constants are provided in those figures for each conditions tested. In this section, we focus on the ratio of apparent oxidation reaction rate constants for sulfolane versus acetonitrile solvent, with the experimental values presented in Table 2. Using the ratios, rather than absolute rate constants, eliminates effects of “better” versus “worse” Ti active sites, and it places the focus on the pore environment and how it controls access by the reactant to the active site in different solvents. As shown in Table 2, the reference case of 1‐phenylethanol and Ti–SiO_2_‐A has a reaction rate in sulfolane 5.6 times that of acetonitrile. The macroporous catalysts (on the left) generally have sulfolane:acetonitrile ratios >2, while the microporous catalysts have ratios that decrease to 1 and lower. For any given material, rate ratios were generally smaller for larger reactants, although the transition between ‘large’ and ‘small’ reactants depends on the nature of the support, beyond the effect of the average pore diameter. For example, with Ti–SiO_2_‐A, the tolylethanol reactant behaves like the naphthylethanol reactant, while for Ti–SiO_2_‐C, the tolylethanol reactant is indistinguishable from 1‐phenylethanol. We also note that for the TS‐1 catalyst, the apparent rates for sulfolane became smaller than those with acetonitrile (ratio < 1). To us, ratios < 1 indicate that the rate of diffusive transport of the sulfolane–alcohol complex to the active sites has become dominant, and the large size (slow diffusion) of the sulfolane–alcohol complex slows rates accordingly. We exclude the alternate interpretation, which is that the decreasing ratios indicate decreasing roles for the reactant–solvent complex in pores, and specifically at the transition state. This would require relative rates to converge to a value of 1 from above, which is not observed. The other piece of evidence that implicates a tradeoff between slow diffusion and faster intrinsic kinetics in sulfolane is a comparison of Ti–SiO_2_‐A and Ti–SiO_2_‐C. The two supports have very similar pore size distribution and surface chemistry, but the higher Ti loading and smaller particle size decreases the diffusion limitation in Ti–SiO_2_‐A, leading to a higher ratio of apparent oxidation reaction rate constants.

**TABLE 2:** Ratios of the initial alcohol oxidation rate constant^a^ with sulfolane solvent versus acetonitrile solvent for catalysts at 80°C (sulfolane: acetonitrile ratios).

	Ti–SiO_2_‐A	Ti–SiO_2_‐C	Hydrophilic Ti‐BEA	Hydrophobic Ti‐BEA	TS‐1
1‐phenylethanol	5.6 ± 0.33	2.5 ± 0.14	1.4 ± 0.10	1.5 ± 0.10	0.9 ± 0.07
1‐(p‐tolyl)ethanol	2.1 ± 0.15	2.8 ± 0.26	N.D.	N.D.	0.6 ± 0.02
1‐(2‐naphthyl)ethanol	2.0 ± 0.18	1.3 ± 0.10	N.D.	N.D.	0.7 ± 0.13

a Errors represent one standard error of the sample mean.

### 3.4 Local Active Site Effects

Previous studies on epoxidation reactions in confined cavities, such as in BEA and MFI zeolites or in silica‐overcoated materials suggested that the interplay between the local chemistry (e.g., hydrophilicity or SiOH density) and the local confinement could affect the rate of reaction [19, 24, 25, 29, 31, 40, 42]. It was pointed out that layers of hydrogen‐bonding solvent around the silica framework could mediate the interaction between silica framework and reactant molecules and consequently increase the reaction rate by lowering activation energy [26]. In order to study this effect in Ti‐BEA materials, 1‐phenylethanol oxidation reaction was performed with both hydrophilic (SiOH‐rich) Ti‐BEA and hydrophobic (SiOH‐poor) Ti‐BEA catalysts of the same average particle size and Ti loading, and the results were reported in Table 3. The absolute reaction rate constants for both solvents were higher in the hydrophobic BEA zeolite, reflecting intrinsic differences in the active sites of the two Ti‐BEA catalysts due to differences in their synthesis. To eliminate the effect of intrinsic activities in favor of how the surface chemistry impacts the solvent‐reactant interactions within the pore, we calculated the rate constant ratios of sulfolane versus acetonitrile (as done for Table 2). The experimental data showed no difference between the ratios with hydrophilic and hydrophobic Ti‐BEA catalyst. With the single‐component diffusion coefficient of 1‐phenylethanol assumed to be 10^−14 ^m^2^/s [38], the Weisz–Prater parameters were estimated to be of order 10^−3^–10^−2^, which for microporous materials indicates strong diffusion limitation. This is because, when the kinetic diameter of the reactant is on the same order of magnitude as the pore size of the zeolite, the adsorption of other species within the zeolite micropores significantly reduces the effective diffusion coefficient of the reactant below the single‐component diffusion coefficient, causing the reaction to be in strongly diffusion‐limited regime even with a Weisz–Prater parameter on the order 10^−5^–10^−3^ [43]. As a result, under the reaction conditions of this study, differences in the ratios of reaction rate constants were not driven by local hydrophilicity of the active site environment, but instead controlled by transport of alcohol reactants and the solvent through reactant–solvent complexes.

**TABLE 3:** 1‐Phenylethanol oxidation rate constant for Ti‐BEA catalysts and overcoated SiO_2_@(Ti–SiO_2_) catalysts at 80°C.

Catalyst	Acetonitrile solvent^a^	Sulfolane solvent^a^	Sulfolane/acetonitrile^b^
Hydrophilic Ti‐BEA	18. ± 1.0	24. ± 1.3	1.4 ± 0.10
Hydrophobic Ti‐BEA	38. ± 2.2	56. ± 1.7	1.5 ± 0.10
2cFO SiO_2_@(Ti–SiO_2_)	7.5 ± 0.7	11. ± 0.7	1.4 ± 0.15
10cFO SiO_2_@(Ti–SiO_2_)	4.2 ± 0.5	14. ± 0.8	3.4 ± 0.45
2cPO SiO_2_@(Ti–SiO_2_)	10. ± 1.1	15. ± 0.3	1.5 ± 0.17
10cPO SiO_2_@(Ti–SiO_2_)	3.8 ± 0.3	21. ± 0.6	5.4 ± 0.49

a Reaction rate constants in units of L/mol Ti/hr.

b Dimensionless ratio of reaction rate constants. Error bars represent one standard error of the sample mean.

The above results show that exploiting local pore environment effects is challenging in the face of strong diffusional limitations that may run counter to effects on transition state stabilization. Therefore, local effects were further investigated using overcoated SiO_2_@(Ti–SiO_2_) catalysts. These catalysts have wide pores except immediately surrounding the active site, and they should therefore be less susceptible to overwhelming transport effects than the BEA samples. All the reactions performed showed a linear relationship between turnover and reaction time, as seen in Figure S11. The reaction rate constants were shown in Table 3. The Weisz–Prater parameters for all reactions with overcoated catalysts were smaller than 0.85, whereas the typical Weisz–Prater parameter for reactions with macroporous catalysts were greater than 1.5, as shown in Table S1. Hence, the diffusion effect was somewhat smaller with overcoated catalysts and the apparent reaction rate constants could well reflect the rate of reaction. For experiments with acetonitrile solvent, the 2cPO material showed the highest reaction rate, 2cFO material showed the second highest rate, and the two 10‐cycle materials showed comparable, low rates. These trends were identical to a previous study on limonene epoxidation reaction in acetonitrile with the same catalysts [28], indicating that they could reflect the intrinsic activity of these overcoated catalysts.

When the solvent was changed to sulfolane, rates over 2cFO and 2cPO were 1.5 times that of the acetonitrile solvent, while rates over 1 cFO or 10cPO increased dramatically to 3–5 times that of the acetonitrile solvent. For the 2‐cycle materials, the increase was much smaller than that with Ti–SiO_2_‐A (5.6 times) and Ti–SiO_2_‐C (2.5 times), which we explain by the steric confinement around titanium site made by the silica overcoat. For the 10‐cycle materials, the large rate constant ratios with sulfolane over acetonitrile comes from a decrease in absolute rates in acetonitrile coupled to an increase in absolute rates in sulfolane, as compared to those of the 2‐cycle materials. In all of these materials, the overcoating process removes some active sites. The loss of active sites is apparent in the low absolute rates with 10‐cycle materials in acetonitrile. However, for the sites that remain accessible, the Ti atoms become increasingly surrounded by an OH‐rich cavity akin to a hydrophilic zeolite pore. These silanol defects stabilize the hydrogen‐bonded networks among solvent and reactant molecules, and consequently lower the reaction free energy [19, 24]. Absent the transport restrictions that exist in bulk zeolites, this effect is particularly pronounced in sulfolane, explaining the large absolute rates and rates relative to acetonitrile for the 10‐cycle overcoated samples.

## 4 Conclusions

The effects of a hydrogen‐bonding solvent (sulfolane) on the apparent rate of catalytic benzylic alcohol oxidation were studied with respect to reactant size, catalyst pore structure, and bulk particle morphology. The observed trends in rate reflect a tradeoff between intrinsic rate enhancements from the H‐bonding solvent and strong effects of diffusion through the catalyst particles. Specifically, we take the ratio of the rate constants in sulfolane versus acetonitrile as an indicator of the roles of the pore and solvent, separate from local active site effects. These ratios decreased with decreasing catalyst pore size and increasing reactant size. Further evidence for rate‐controlling diffusion comes from absolute and relative rates that are also sensitive to particle sizes and Ti atom densities. We interpret these findings to indicate that H‐bonding association between alcohol reactant and sulfolane solvent creates a species with low effective diffusivity through the porous structures of the catalysts. This runs counter to the role of the hydrogen bonding solvent in increasing intrinsic reaction rate constants on non‐ or macroporous catalysts by facilitating proton transfer during the oxygen transfer step.

Given the strong transport restrictions possible for these reactions in porous oxides, we sought an alternate strategy to alter intrinsic reactivity at the active sites. Previously demonstrated silica overcoats around the titanium active sites created hydrophilic, sterically confined environments, which gave pronounced rate increases in the sulfolane solvent on a relative and absolute basis. Absolute rates over these overcoated materials approached those over Ti‐BEA, which tend to be class‐leading materials for catalytic oxidation. Serendipitously, it was also demonstrated that these overcoats help stabilize the catalyst over long storage periods. Overall, this study demonstrated that the complex interplay among solvent, reactant, porous structure of catalyst, and chemical environment around the catalytic site can be leveraged to give large changes in reaction rate due to both intrinsic kinetics and diffusional control. Full consideration of these effects may allow better control over selective oxidation cascade reactions by, for example, selectively accelerating the first reaction in a series, while inhibiting the second.

## Funding

This study was supported by Basic Energy Sciences (DE‐FG02‐03ER15457) and Northwestern University.

## Conflicts of Interest

The authors declare no conflicts of interest.

## Supporting information

Supplementary Material

## Data Availability

The data that supports the findings of this study are available in the supplementary material of this article.
