# Supplementary material for: Effect of Solvent Hydrogen‐Bonding and Catalyst Pore Size on Catalytic Oxidation of Benzylic Alcohols
Source: Chemphyschem. 2026 Jul 31;27(15):e70471. doi: 10.1002/cphc.70471 (PMC13425448; doi:10.1002/cphc.70471)
Supplement: Supplementary file 1 — Supplementary Material [file CPHC-27-e70471-s001.pdf]

Supporting Information

for

Effect of Hydrogen-bonding Solvent and Catalyst Pore Size on Catalytic Benzylic Alcohol Oxidation

Dian Jing† and Justin M. Notestein*

Department of Chemical and Biological Engineering, Northwestern University, Evanston, Illinois, USA

** corresponding author. Email: j-notestein@northwestern.edu*

† now at *Department of Physics and Pritzker School of Molecular Engineering, University of Chicago, Chicago, Illinois, USA*

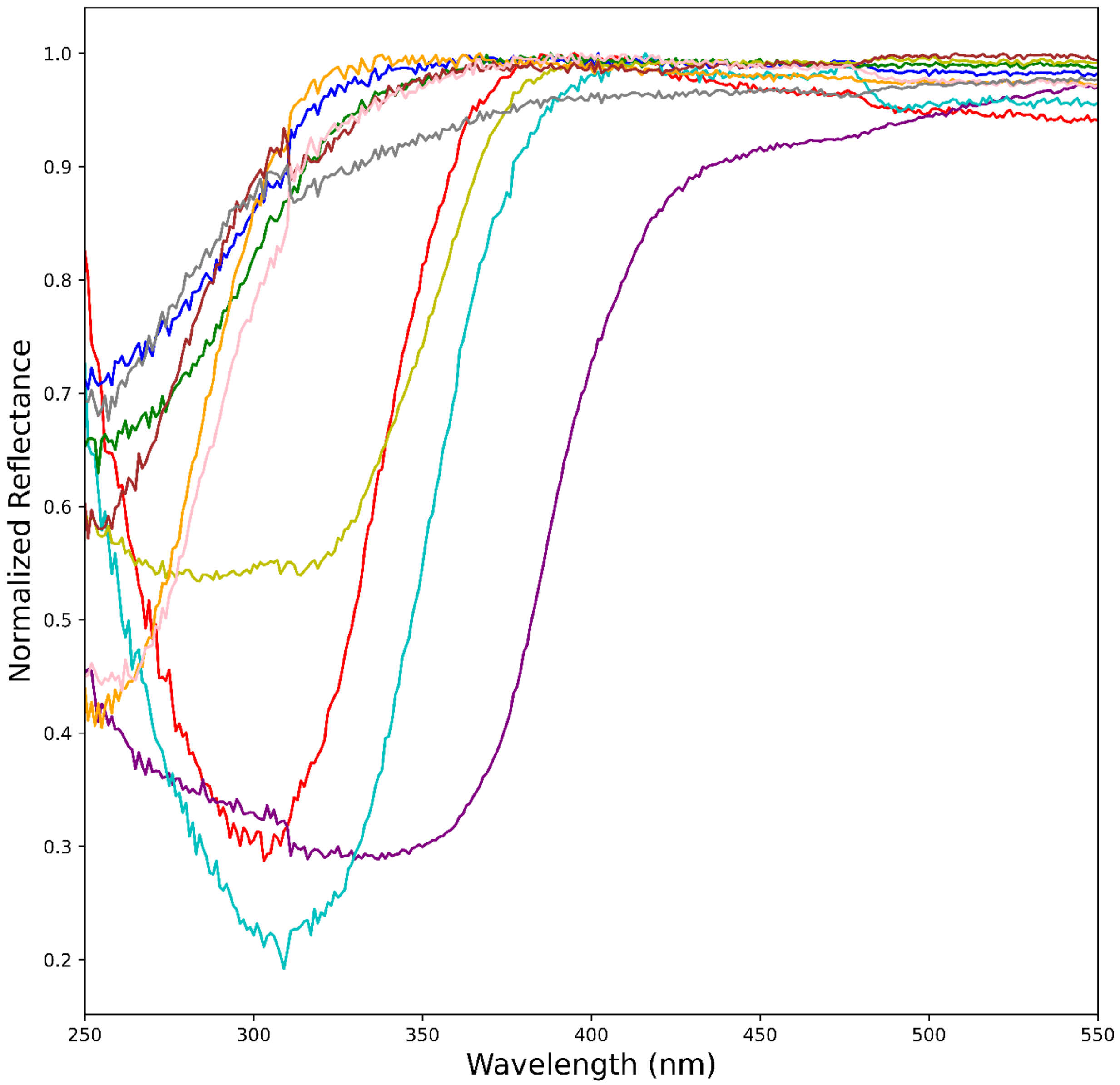

Figure S1. DRUV-vis spectra for Ti-SiO_2-A (red), Ti-SiO_2-C (cyan), hydrophilic Ti-BEA (blue), hydrophobic Ti-BEA (green), TS-1 (yellow), overcoated 2 cFO SiO_2-Ti-SiO_2 (orange), 10 cFO SiO_2-Ti-SiO_2 (brown), 2 cPO SiO_2-Ti-SiO_2 (pink), and 10 cPO SiO_2-Ti-SiO_2 (gray). Total reflectance normalized to the highest feature.

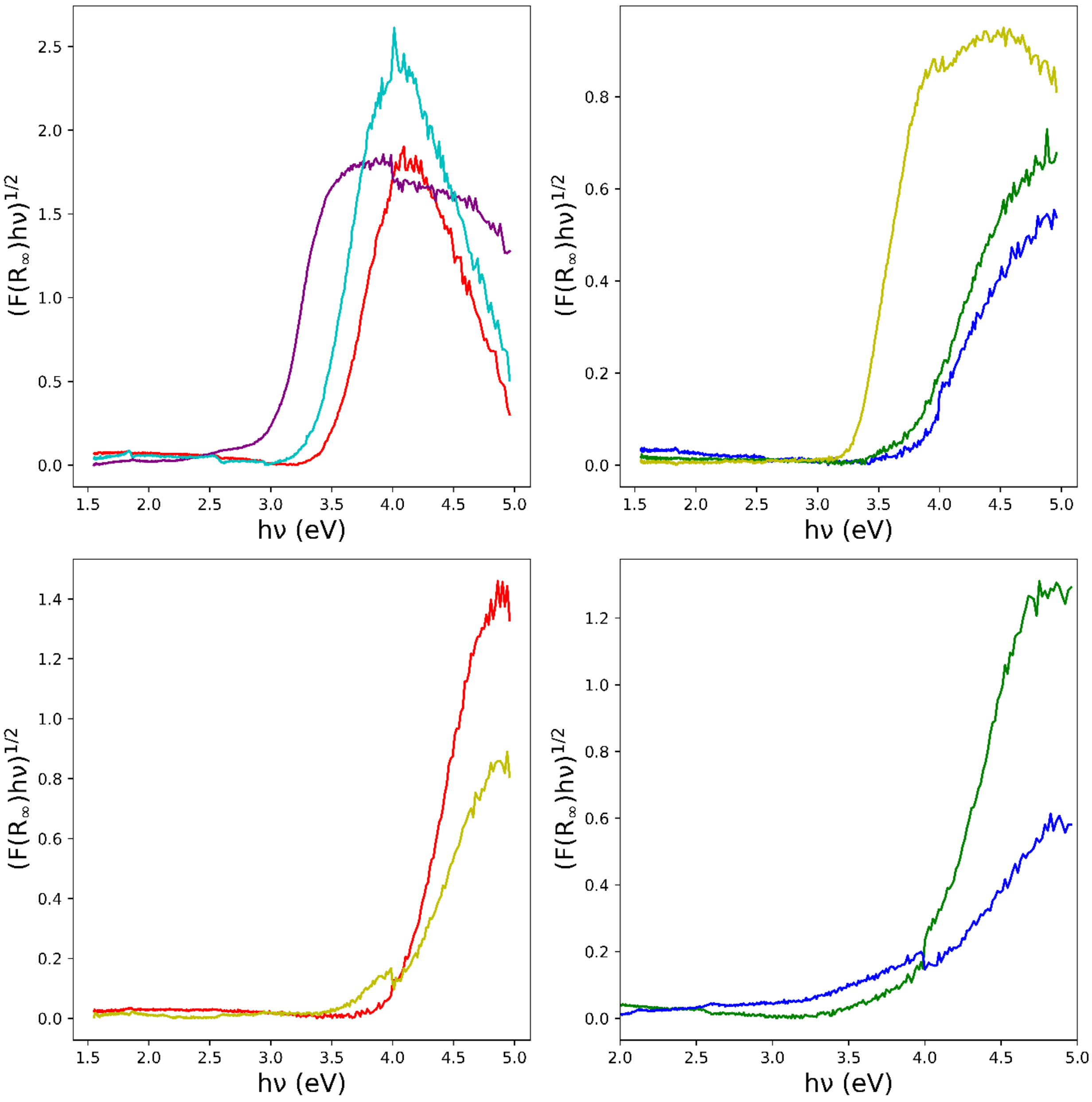

Figure S2. The indirect transition Tauc plots of the DRUV-vis spectra for Ti-SiO_2-A (top left, red), Ti-SiO_2-B (top left, purple), Ti-SiO_2-C (top left, cyan), hydrophilic Ti-BEA (top right, blue), hydrophobic Ti-BEA (top right, green), TS-1 (top right, yellow), overcoated 2 cFO SiO_2-Ti-SiO_2 (bottom left, red), 10 cFO SiO_2-Ti-SiO_2 (bottom left, yellow), 2 cPO SiO_2-Ti-SiO_2 (bottom right, green), and 10 cPO SiO_2-Ti-SiO_2 (bottom right, blue).

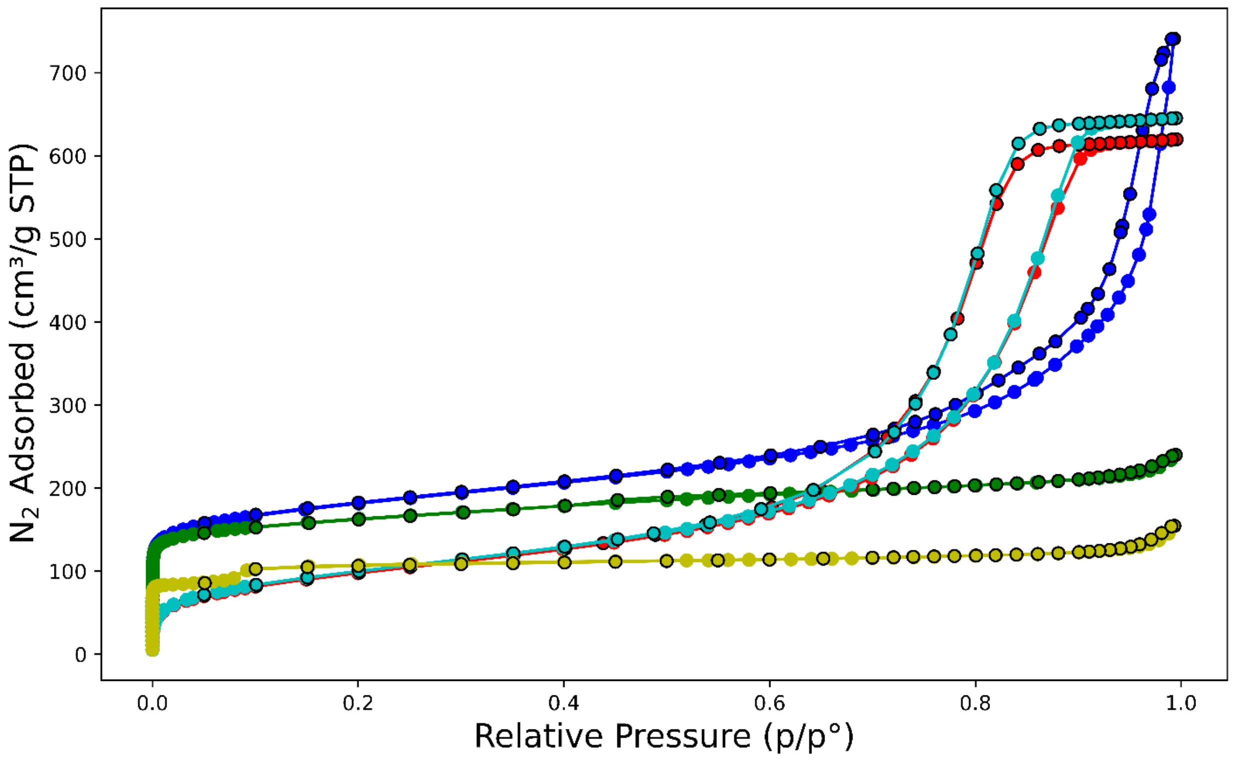

Figure S3. N_2 physisorption isotherms obtained at -196 °C for Ti-SiO_2-A (red), Ti-SiO_2-C (cyan), hydrophilic Ti-BEA (blue), hydrophobic Ti-BEA (green), and TS-1 (yellow). Desorption points are labeled with a black border.

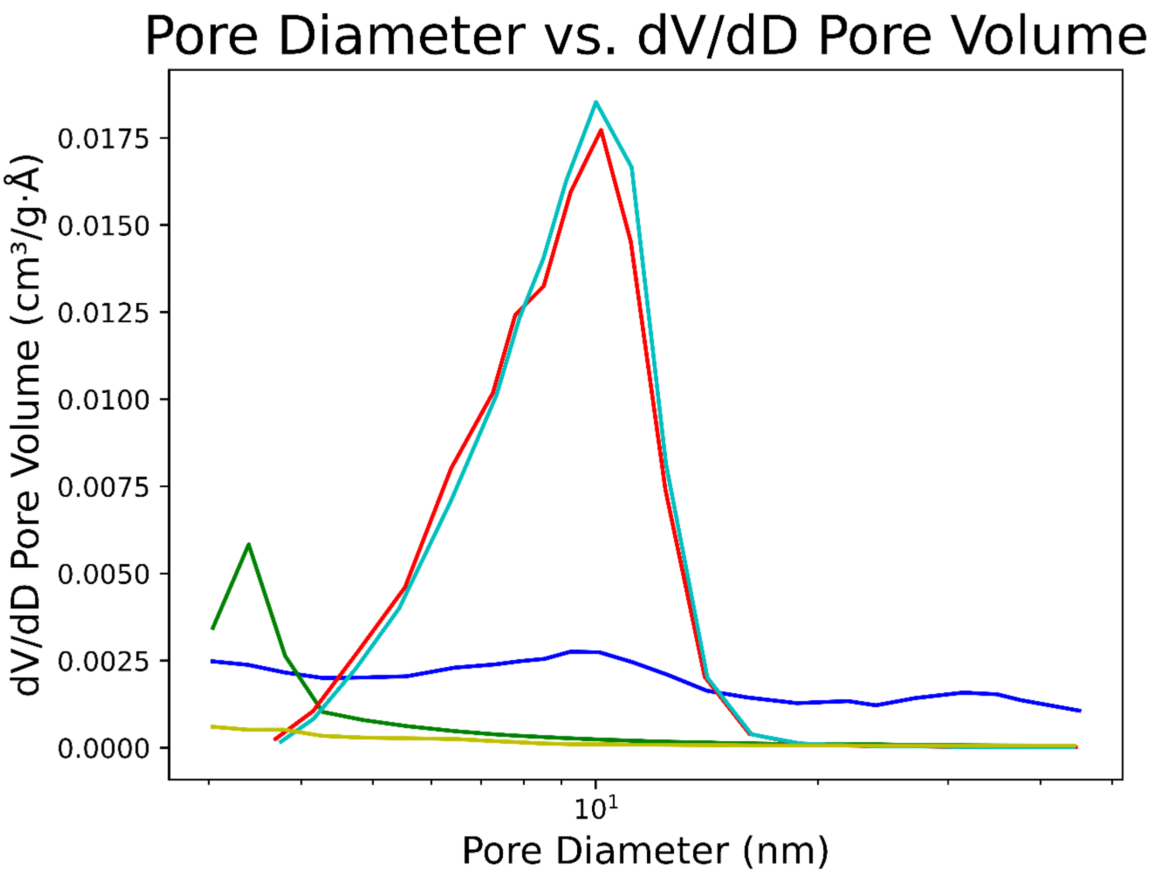

Figure S4. BJH pore size distributions for Ti-SiO_2-A (red), Ti-SiO_2-C (cyan), hydrophilic Ti-BEA (blue), hydrophobic Ti-BEA (green), and TS-1 (yellow) based on the desorption branch of N_2 physisorption isotherms obtained at -196 °C. Ti-BEA materials and TS-1 have regular, crystalline structures with 0.67 nm pores and 0.5 nm pores, respectively.

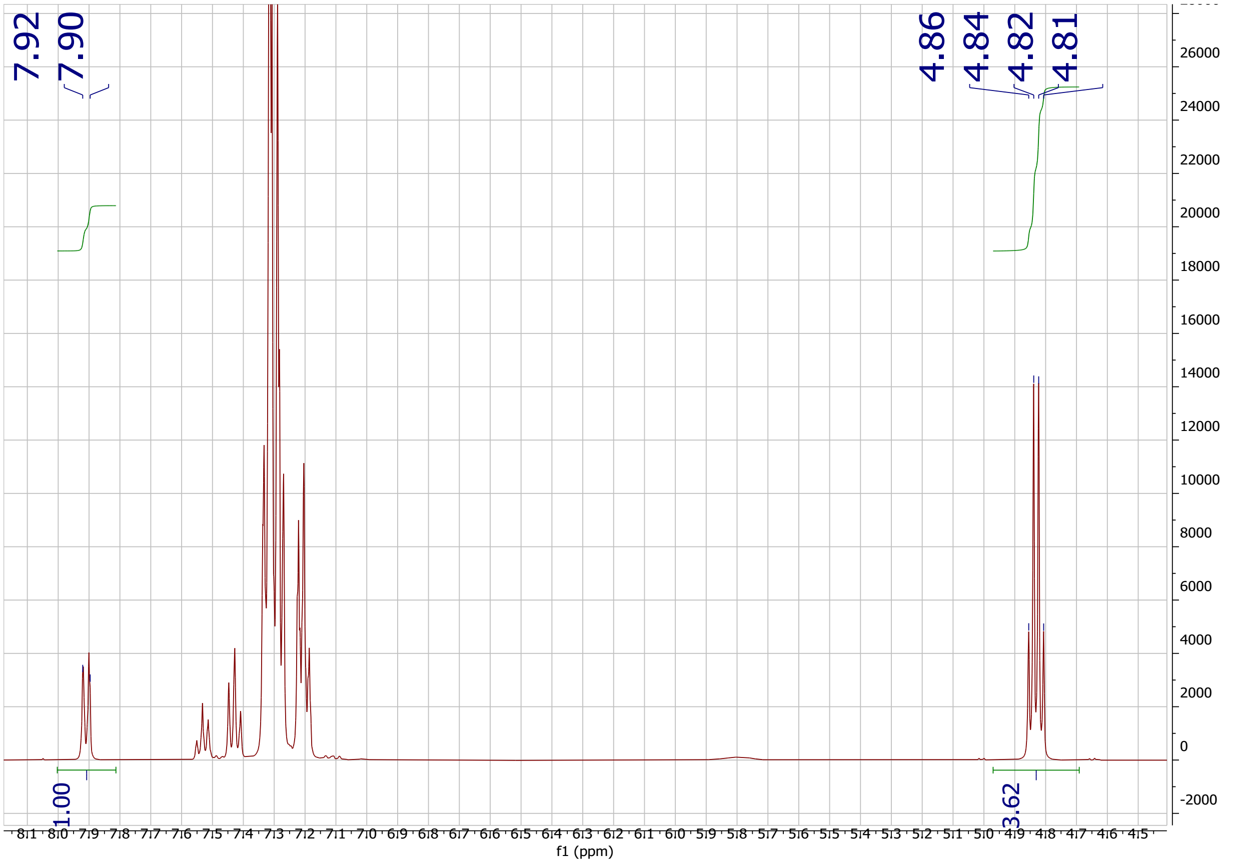

Figure S5. Representative NMR spectrum for reaction sample taken after 8 hours of reaction. The quartet peak at 4.83 ppm from 1-phenylethanol and the doublet peak at 7.91 ppm from acetophenone were used to quantify the concentration of both species. Reaction conditions: 80 °C, 43 mg of TS-1 catalyst (16 µmol Ti), 8.0 mmol 1-phenylethanol, 20 mmol H_2O_2, 10 mL acetonitrile, shaking at 500 rpm. NMR conditions: 32 scans, 5 seconds of D1 delay, sample from reactor diluted 1:10 in $CDCl_3$ solvent, solvent suppression for acetonitrile applied.

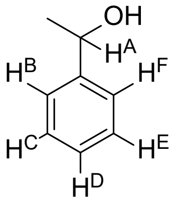

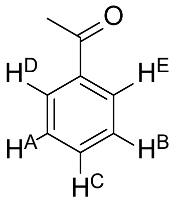

^{1}H NMR (400 MHz, $CDCl_3$) peak identification:

1-phenylethanol: δ 4.83 (q, 1H, H^A), 7.18-7.34 (m, 5H, H^{B-F}) ppm.

Acetophenone: δ 7.43 (t, 2H, H^{A-B}), 7.53 (t, 1H, H^C), 7.91 (d, 2H, H^{D-E}) ppm.

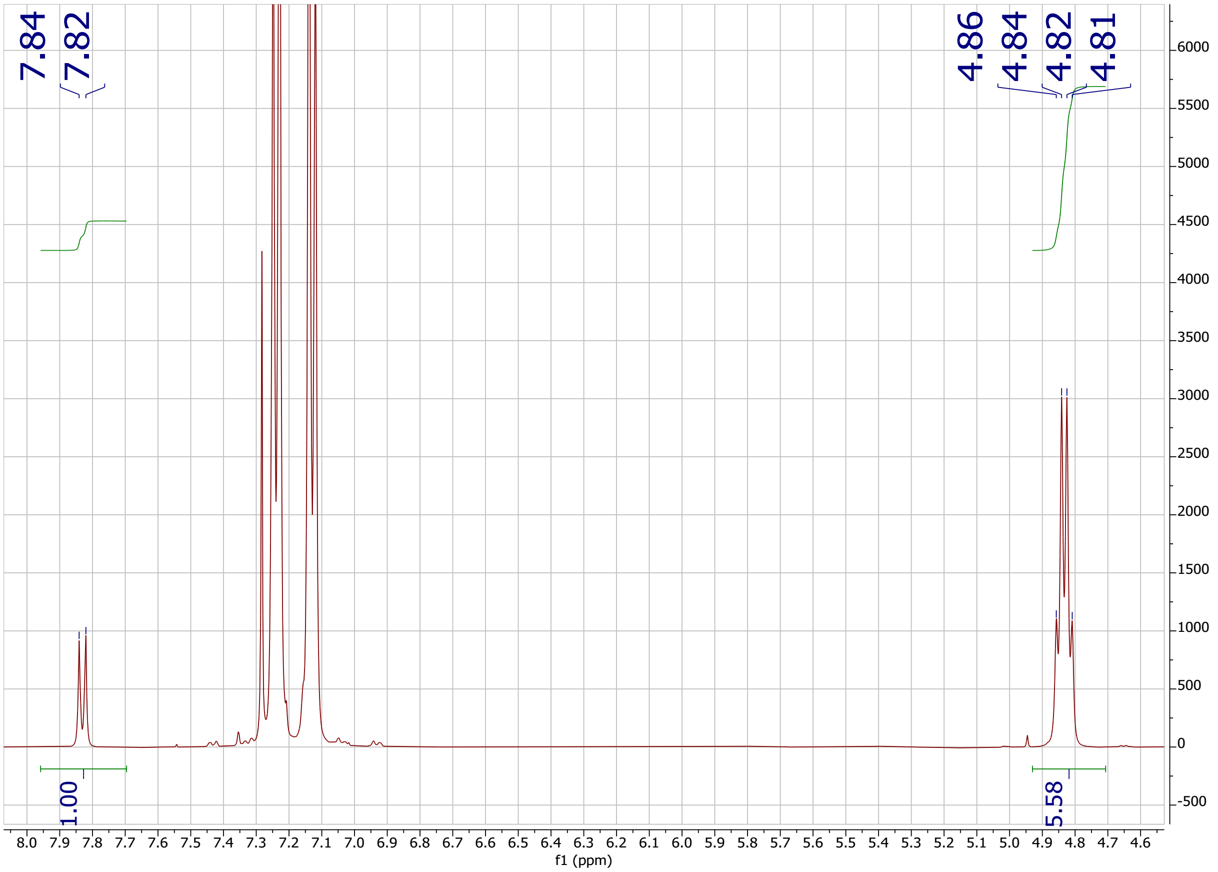

Figure S6. Representative NMR spectrum for reaction sample taken after 8 hours of reaction. The quartet peak at 4.83 ppm from 1-(p-tolyl)ethanol and the doublet peak at 7.83 ppm from 4'-methylacetophenone were used to quantify the concentration of both species. Reaction conditions: 80 °C, 43 mg of TS-1 catalyst (16 µmol Ti), 7.0 mmol 1-(p-tolyl)ethanol, 20 mmol H_2O_2, 10 mL acetonitrile, shaking at 500 rpm. NMR conditions: 32 scans, 5 seconds of D1 delay, sample from reactor diluted 1:10 in $CDCl_3$ solvent, solvent suppression for acetonitrile applied.

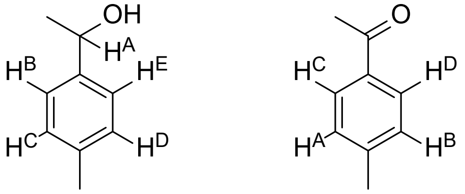

^{1}H NMR (400 MHz, $CDCl_3$) peak identification:

1-(p-tolyl)ethanol: δ 4.83 (q, 1H, H^A), 7.12-7.28 (m, 4H, H^{B-E}) ppm.

4'-methylacetophenone: δ 7.12-7.28 (m, 2H, H^{A-B}), 7.83 (d, 2H, H^{C-D}) ppm.

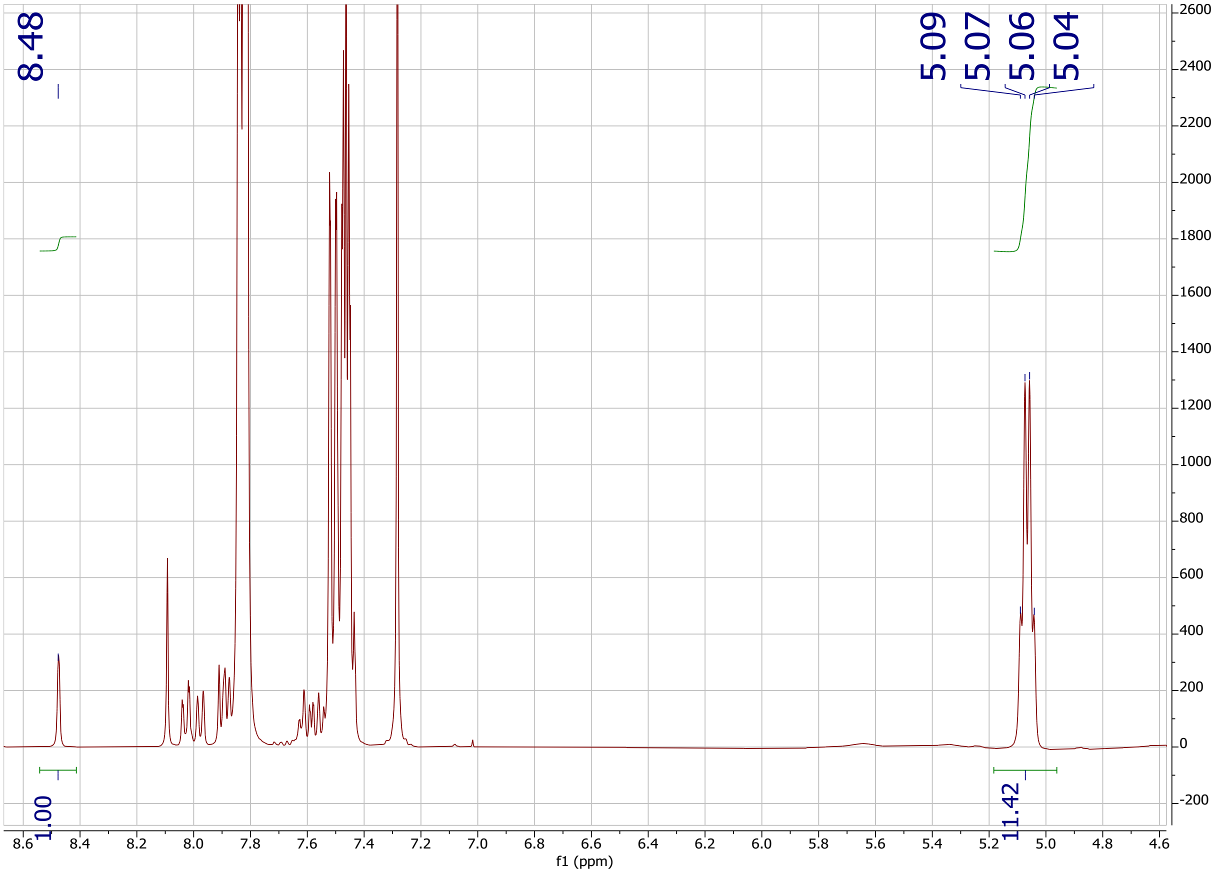

Figure S7. Representative NMR spectrum for reaction sample taken after 8 hours of reaction. The quartet peak at 5.07 ppm from 1-(2-naphthyl)ethanol and the singlet peak at 8.48 ppm from 2-acetylnaphthalene were used to quantify the concentration of both species. Reaction conditions: 80 °C, 43 mg of TS-1 catalyst (16 µmol Ti), 5.7 mmol 1-(2-naphthyl)ethanol, 20 mmol H_2O_2, 10 mL acetonitrile, shaking at 500 rpm. NMR conditions: 32 scans, 5 seconds of D1 delay, sample from reactor diluted 1:10 in $CDCl_3$ solvent, solvent suppression for acetonitrile applied.

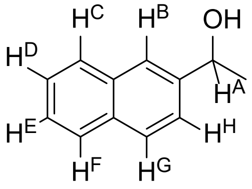

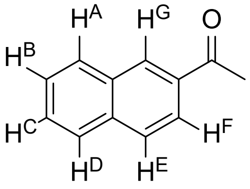

NMR peak identifications:

1-(2-naphthyl)ethanol: δ 5.07 (q, 1H, H^{A}), 7.28-8.09 (m, 7H, $H^{B\text{-}H}$) ppm.

2-acetylnaphthalene: δ 7.28-8.09 (m, 6H, $H^{A\text{-}F}$), 8.48 (s, 1H, H^{G}) ppm.

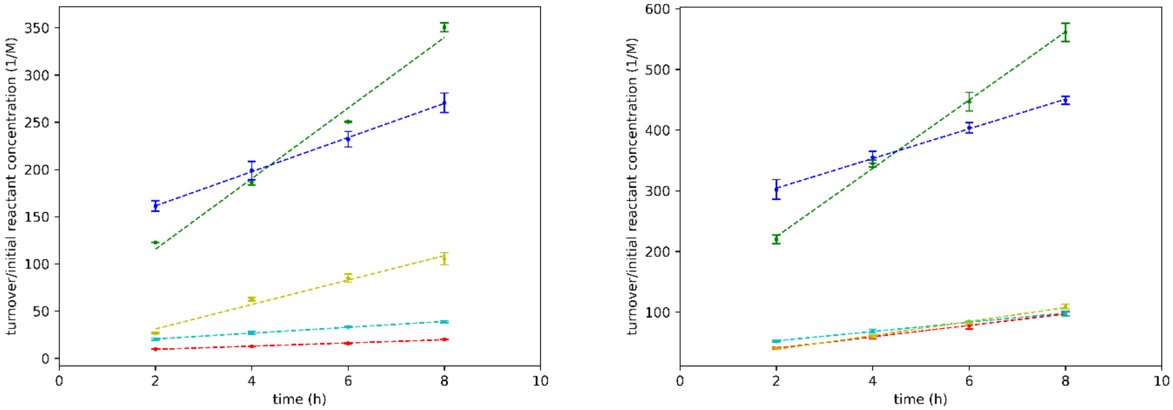

Catalyst	Acetonitrile (L/mol Ti/hr)	Sulfolane (L/mol Ti/hr)	Sulfolane : acetonitrile ratio
Ti-SiO2-A	1.7 ± 0.1	9.4 ± 0.4	5.6 ± 0.33
Ti-SiO2-C	3.1 ± 0.1	7.7 ± 0.3	2.5 ± 0.14
Hydrophilic Ti-BEA	18. ± 1.0	24. ± 1.3	1.4 ± 0.10
Hydrophobic Ti-BEA	38. ± 2.2	56. ± 1.7	1.5 ± 0.10
TS-1	13. ± 0.9	11. ± 0.4	0.9 ± 0.07

Figure S8. Plots of 1-phenylethanol turnover divided by the initial 1-phenylethanol concentration as a function of reaction time with acetonitrile solvent (left panel) and sulfolane solvent (right panel) catalyzed by Ti-SiO_2-A (red), Ti-SiO_2-C (cyan), hydrophilic Ti-BEA (blue), hydrophobic Ti-BEA (green), and TS-1 (yellow). The slopes of the regression lines correspond to the reaction rate constant, which are normalized by the amount of titanium atoms and tabulated. The nonzero y-intercept at zero time reflects the reaction initiation effect. Error bars represent one standard error of the sample mean. Conditions: 80 °C, 8.0 mmol 1-phenylethanol, 20 mmol H_2O_2, 10 mL solvent, shaking at 500 rpm. Catalyst: 90 mg of Ti-SiO_2-A catalyst (28 µmol Ti), or 100 mg of Ti-SiO_2-C catalyst (16 µmol Ti), or 50 mg of hydrophilic Ti-BEA (3.1 µmol Ti), or 156 mg of hydrophobic Ti-BEA (9.8 µmol Ti), or 43 mg of TS-1 (16 µmol Ti).

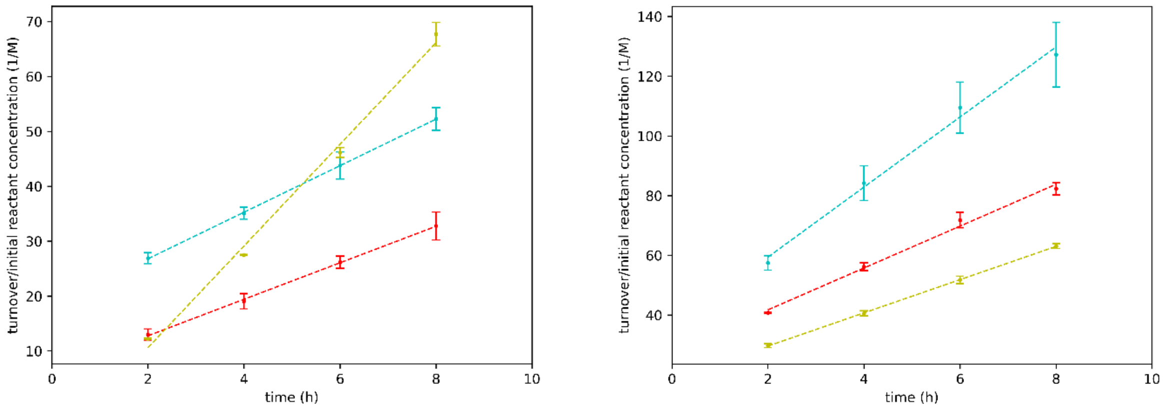

Catalyst	Acetonitrile (L/mol Ti/hr)	Sulfolane (L/mol Ti/hr)	Sulfolane : acetonitrile ratio
Ti-SiO_2-A	3.3 ± 0.2	7.0 ± 0.3	2.1 ± 0.15
Ti-SiO_2-C	4.2 ± 0.2	12. ± 0.9	2.8 ± 0.26
TS-1	9.2 ± 0.3	5.6 ± 0.1	0.6 ± 0.02

Figure S9. Plots of 1-(p-tolyl)ethanol turnover divided by the initial 1-(p-tolyl)ethanol concentration as a function of reaction time with acetonitrile solvent (left panel) and sulfolane solvent (right panel) catalyzed by Ti-SiO_2-A (red), Ti-SiO_2-C (cyan), and TS-1 (yellow). The slopes of the regression lines correspond to the reaction rate constant, which are normalized by the amount of titanium atoms and tabulated. The nonzero y-intercept at zero time reflects the reaction initiation effect. Error bars represent one standard error of the sample mean. Conditions: 80 °C, 7.0 mmol 1-(p-tolyl)ethanol, 20 mmol H_2O_2, 10 mL solvent, shaking at 500 rpm. Catalyst: 120 mg of Ti-SiO_2-A catalyst (37 µmol Ti), or 100 mg of Ti-SiO_2-C catalyst (16 µmol Ti), or 43 mg of TS-1 (16 µmol Ti).

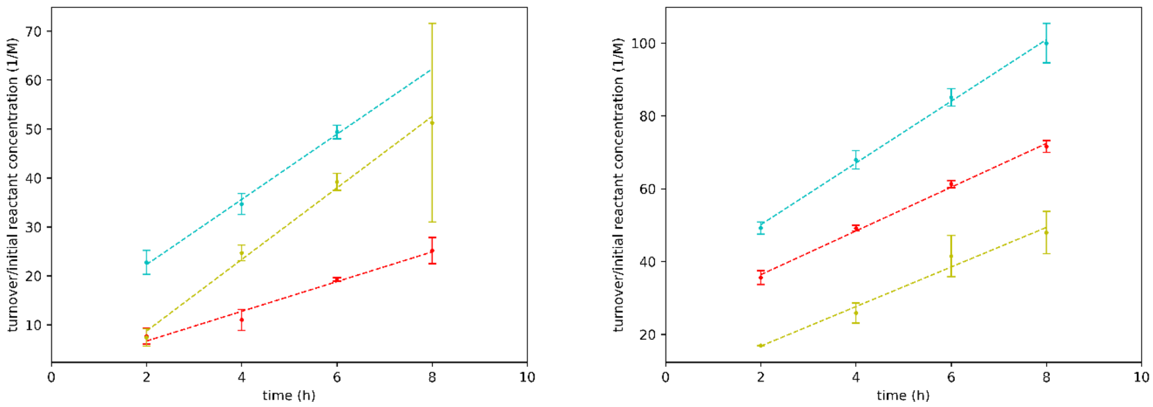

Catalyst	Acetonitrile (L/mol Ti/hr)	Sulfolane (L/mol Ti/hr)	Sulfolane : acetonitrile ratio
Ti-SiO$_2$-A	3.0 ± 0.3	6.0 ± 0.2	2.0 ± 0.18
Ti-SiO$_2$-C	6.7 ± 0.4	8.5 ± 0.4	1.3 ± 0.10
TS-1	7.4 ± 1.1	5.4 ± 0.5	0.7 ± 0.13

Figure S10. Plots of 1-(2-naphthyl)ethanol turnover divided by the initial 1-(2-naphthyl)ethanol concentration as a function of reaction time with acetonitrile solvent (left panel) and sulfolane solvent (right panel) catalyzed by Ti-SiO$_2$-A (red), Ti-SiO$_2$-C (cyan), hydrophilic Ti-BEA (blue), hydrophobic Ti-BEA (green), and TS-1 (yellow). The slopes of the regression lines correspond to the reaction rate constant, which are normalized by the amount of titanium atoms and tabulated. The nonzero y-intercept at zero time reflects the reaction initiation effect. Error bars represent one standard error of the sample mean. Conditions: 80 °C, 5.7 mmol 1-(2-naphthyl)ethanol, 20 mmol H_2O_2, 10 mL solvent, shaking at 500 rpm. Catalyst: 120 mg of Ti-SiO$_2$-A catalyst (37 µmol Ti), or 100 mg of Ti-SiO$_2$-C catalyst (16 µmol Ti), or 43 mg of TS-1 (16 µmol Ti).

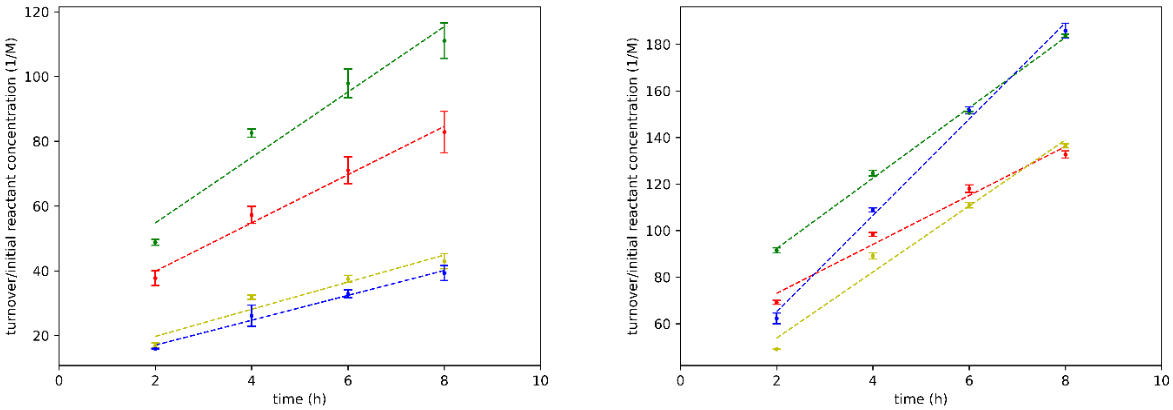

Catalyst	Acetonitrile (L/mol Ti/hr)	Sulfolane (L/mol Ti/hr)	Sulfolane : acetonitrile ratio
2 cFO SiO_2@(Ti-SiO_2)	7.5 ± 0.7	11. ± 0.7	1.4 ± 0.15
10 cFO SiO_2@(Ti-SiO_2)	4.2 ± 0.5	14. ± 0.8	3.4 ± 0.45
2 cPO SiO_2@(Ti-SiO_2)	10. ± 1.1	15. ± 0.3	1.5 ± 0.17
10 cPO SiO_2@(Ti-SiO_2)	3.8 ± 0.3	21. ± 0.6	5.4 ± 0.49

Figure S11. Plots of 1-phenylethanol turnover divided by the initial 1-phenylethanol concentration as a function of reaction time with acetonitrile solvent (left panel) and sulfolane solvent (right panel) catalyzed by overcoated 2 cFO SiO_2-Ti-SiO_2 (red), 10 cFO SiO_2-Ti-SiO_2 (yellow), 2 cPO SiO_2-Ti-SiO_2 (green), and 10 cPO SiO_2-Ti-SiO_2 (blue). The slopes of the regression lines correspond to the reaction rate constant, which are normalized by the amount of titanium atoms and tabulated. The nonzero y-intercept at zero time reflects the reaction initiation effect. Error bars represent one standard error of the sample mean. Conditions: 80 °C, 8.0 mmol 1-phenylethanol, 20 mmol H_2O_2, 10 mL solvent, shaking at 500 rpm. Catalyst: 130 mg of overcoated 2 cFO SiO_2-Ti-SiO_2 catalyst (14 µmol Ti), or 130 mg of overcoated 10 cFO SiO_2-Ti-SiO_2 catalyst (9.0 µmol Ti), or 64 mg of overcoated 2 cPO SiO_2-Ti-SiO_2 (6.9 µmol Ti), or 132 mg of overcoated 10 cPO SiO_2-Ti-SiO_2 (9.1 µmol Ti).

Catalyst	Solvent	Thiele modulus	Weisz-Prater parameter
Ti-SiO_2-A	Acetonitrile	0.52	0.27
	sulfolane	1.3	1.5
Ti-SiO_2-C	Acetonitrile	1.6	2.2
	sulfolane	2.8	5.6
2 cFO SiO_2-Ti-SiO_2	Acetonitrile	0.66	0.42
	sulfolane	0.79	0.59
10 cFO SiO_2-Ti-SiO_2	Acetonitrile	0.39	0.15
	sulfolane	0.73	0.51
2 cPO SiO_2-Ti-SiO_2	Acetonitrile	0.77	0.57
	sulfolane	0.95	0.85
10 cPO SiO_2-Ti-SiO_2	Acetonitrile	0.37	0.14
	sulfolane	0.88	0.74

Table S1. Thiele Modulus and Weisz-Prater parameters for Ti-SiO2-A, Ti-SiO2-C, and over-coated SiO_2-Ti-SiO_2 catalysts. The diffusion coefficient is assumed to be 10^{-12} m^2/s and independent of solvent. The density of catalysts was assumed to be 0.75 g/cm^3 based on typical values of total pore volume obtained by N_2 physisorption.
